# Association of intra-operative adverse events with gall bladder wall thickness in patients undergoing laparoscopic cholecystectomy for acute calculous cholecystitis: A prospective study from a low middle income country

**DOI:** 10.12669/pjms.42.(11AASC).15799

**Published:** 2026-04

**Authors:** Narmeen Asif, Abdul Ahad Sohail, Koonj Sundardas, Abdul Rehman Alvi

**Affiliations:** 1 Dr. Narmeen Asif, Department of Surgery, Aga Khan University Hospital, Karachi, Pakistan; 2 Abdul Ahad Sohail, Department of Surgery, Aga Khan University Hospital, Karachi, Pakistan; 3 Koonj Sundardas, Department of Surgery, Aga Khan University Hospital, Karachi, Pakistan; 4 Abdul Rehman Alvi, Department of Surgery, Aga Khan University Hospital, Karachi, Pakistan

**Keywords:** Acute calculous cholecystitis, Difficult cholecystectomy, Gallbladder wall thickness, Intraoperative adverse events, Laparoscopic cholecystectomy, Preoperative risk stratification

## Abstract

**Objective:**

Acute calculous cholecystitis is one of the most common surgical infectious disease emergencies worldwide. Early laparoscopic cholecystectomy (LC) is the definitive treatment, but severe inflammation often leads to technical difficulty and increased intraoperative adverse events. This study evaluated preoperative gallbladder wall thickness (GWT) on ultrasound as a simple, objective predictor of difficult LC in acute calculous cholecystitis.

**Methodology:**

A prospective cross-sectional study was conducted in the Department of Surgery, Aga Khan University Hospital, Karachi, from May to November 2022. Consecutive adult patients (18–60 years) with ultrasound-confirmed acute calculous cholecystitis undergoing LC within 96 hours of symptom onset were included (n=116). GWT was classified as normal (≤3 mm), moderate (3.1–7 mm), and severe (>7 mm). Intraoperative adverse events recorded were operative time >90 minutes, distended gallbladder requiring aspiration, dense adhesions, conversion to open procedure, and drain placement.

**Results:**

Mean GWT was 3.44 ± 1.79 mm. Severe thickening (>7 mm) was present in six patients (5.2%) and was highly significantly associated with prolonged operative time (p<0.001), need for gallbladder aspiration (p<0.001), conversion to open cholecystectomy (p<0.001), and subhepatic drain placement (p<0.001). Dense adhesions showed a strong trend (p=0.069).

**Conclusion:**

Severe preoperative gallbladder wall thickening (>7 mm) is a powerful, reproducible marker of intense inflammation and can predicts difficult laparoscopic cholecystectomy in acute infectious cholecystitis. Routine reporting of GWT allows accurate risk stratification, facilitates early involvement of experienced surgeons, reduces conversion rates, and optimizes outcomes in this common infectious surgical emergency – making it a practical and cost-effective surgical solution, especially in low-middle-income settings.

## INTRODUCTION

Laparoscopic cholecystectomy (LC) is the most performed surgical procedure worldwide and remains the gold standard treatment for symptomatic gallstones and acute calculous cholecystitis.^1^-^4^ Early LC performed within 72–96 hours of symptom onset in acute cholecystitis, which is often referred to as the “golden period”, is associated with lower rates of intraoperative complications, reduced conversion to open surgery, shorter operative time, and decreased hospital stay.^5^-^8^

Despite adherence to this early timing, a significant proportion of cases remain technically difficult due to severe inflammation or adhesions.^3^,^9^ Numerous preoperative risk factors for difficult LC and conversion to open procedure have been identified, including male gender, advanced age, obesity, previous abdominal surgery, elevated white cell count, deranged liver function tests, previous endoscopic retrograde cholangio pancreatography (ERCP), presence of peri-cholecystic fluid and acute cholecystitis itself.^2^-^4^,^6^,^7^,^9^-^11^ Among these, gallbladder wall thickness (GWT) measured on preoperative ultrasonography has emerged as one of the most reliable, objective, and easily obtainable predictors of intraoperative difficulty.^1^,^12^-^15^

Multiple recent studies have consistently demonstrated that GWT >3–5 mm is strongly associated with prolonged operative time, increased bleeding, gallbladder perforation, dense adhesions, need for aspiration, higher conversion rates, and greater overall intraoperative adverse events.^1^,^2^,^12^,^13^,^15^-^17^Severe thickening (>5 mm) reflects intense inflammatory changes and oedema, making dissection in Calot’s triangle hazardous and increasing the risk of bile duct injury.^1^,^2^

In resource-constrained and low-middle-income settings, where senior laparoscopic expertise may not always be immediately available, accurate preoperative identification of potentially difficult cases is crucial for appropriate case selection, timely involvement of experienced surgeons, theatre scheduling, surgical planning, risk stratification and informed patient counselling.^18^,^19^

Although several studies worldwide have validated GWT as a predictor of difficult LC, very limited contemporary data exist from Pakistan and the South Asian region. This prospective study was therefore designed to evaluate the association between preoperative gallbladder wall thickness on ultrasound and the occurrence of intraoperative adverse events in patients undergoing early laparoscopic cholecystectomy for acute calculous cholecystitis at a tertiary-care university hospital in a low-middle-income country.

## METHODOLOGY

This prospective observational study was conducted in the Department of Surgery, Aga Khan University Hospital, Karachi, Pakistan, from May 26 to November 28, 2022.

### Exemption from Ethics Committee:

As the study involved no direct patient interaction and utilized only existing clinical, radiological, and operative records, exemption was granted by the Ethical Review Committee of Aga Khan University (ERC Exemption Ref: 2022-7621-21970; dated July 20, 2022).

Consecutive adult patients (aged 18–70 years) diagnosed with acute calculous cholecystitis who presented within 96 hours of symptom onset and underwent laparoscopic cholecystectomy during the study period, and who had a preoperative abdominal ultrasound performed and reported by a consultant radiologist at Aga Khan University Hospital within 48 hours prior to surgery and reported as Acute Cholecystitis.

Patients with prior biliary surgery, suspected gallbladder malignancy, concomitant choledocholithiasis, cholangitis, pancreatitis, gallstone ileus, pregnancy, bleeding disorders, chronic liver disease, chronic kidney disease, ASA grade >II, those requiring percutaneous cholecystostomy, planned open cholecystectomy, or laparoscopic cholecystectomy performed as part of another major procedure were excluded.

Gallbladder wall thickness (GWT) was measured and reported by consultant radiologists and categorized as normal (≤3 mm), moderate (3.1–7 mm), and severe (>7 mm).

All laparoscopic cholecystectomies were performed or directly supervised by experienced consultant surgeons. Intraoperative adverse events prospectively recorded from operative notes included: operative time >90 minutes, dense adhesions, distended gallbladder requiring aspiration, gallbladder perforation, significant bleeding, bile or stone spillage, subhepatic drain placement, conversion to open cholecystectomy, bile duct injury, and bowel injury. Postoperative hospital stay was also recorded.

Sample size was calculated using OpenEpi version 3.1. Based on a reported conversion rate of 7.8% in difficult cases, a minimum of 111 patients was required (95% CI, 5% margin of error). A total of 116 patients met the inclusion criteria and were enrolled.

### Data analysis:

It was analyzed using SPSS version 24. Continuous variables are expressed as mean ± SD or median (IQR); categorical variables as frequencies and percentages. Associations between GWT categories and intraoperative outcomes were assessed using Chi-square or Fisher’s exact test for categorical variables and Kruskal-Wallis test for continuous variables. A p-value <0.05 was considered statistically significant.

## RESULTS

A total of 116 patients with acute calculous cholecystitis who underwent early laparoscopic cholecystectomy were included. Mean age was 45.5 ± 13.2 years with a marked female predominance (79, 68.1%). Mean BMI was 29.2 ± 5.1 kg/m², with 46 patients (39.7%) being obese (BMI >30 kg/m²). Hypertension (37.9%) and previous abdominal surgery (38.8%) were the most frequent comorbidities (Table-I).

**Table-I:** Demographics and Co-Morbidities of patients included in the study.

Characteristic	Value
***Demographics***	
Mean age (years)	45.5 ± 13.2
Female sex, n (%)	79 (68.1)
Male sex, n (%)	37 (31.9)
***Anthropometrics***	
Mean BMI (kg/m²)	29.2 ± 5.1
***Comorbidities***	
Hypertension, n (%)	44 (37.9)
Diabetes mellitus, n (%)	20 (17.2)
Previous surgery, n (%)	45 (38.8)
Ischemic heart disease, n (%)	4 (3.4)
Asthma, n (%)	3 (2.6)

The mean radiological gallbladder wall thickness (GWT) was 3.44 ± 1.79 mm. Based on ultrasound, 76 (66%) patients had normal GWT, 34 (29%) had moderate thickening, and 6 (5.2%) had severe thickening (Table-II).

**Table-II:** Distribution of gallbladder wall thickness.

GWT Category	Patients (n, %)
Normal (<3 mm)	76 (66.0%)
Moderate (3–7 mm)	34 (29.0%)
Severe (>7 mm)	6 (5.2%)

Overall conversion rate to open cholecystectomy was 3.4% (n=4). The most common intraoperative findings were dense adhesions (55.2%), significant bleeding (25.9%), distended gallbladder requiring aspiration (13.8%), gallbladder perforation (10.3%), bile leak (8.6%), and subhepatic drain placement (11.2%). No bile duct injuries or visceral injuries occurred (Table-III).

**Table-III:** Comparison of gallbladder wall thickness with intraoperative findings (n=116)

Intraoperative findings	Normal GWT	Moderate GWT	Severe GWT	p-value
Intraoperative bile leak	68 (64.2%)	32 (30.2%)	6 (5.7%)	0.538
Stone spillage	73 (65.2%)	34 (30.4%)	5 (4.5%)	0.110
Bile duct injury	0 (0%)	0 (0%)	0 (0%)	–
Bleeding	58 (67.4%)	24 (27.9%)	4 (4.7%)	0.746
Placement of surgical drain	74 (71.8%)	25 (24.3%)	4 (3.9%)	<0.001
Bowel injury	0 (0%)	0 (0%)	0 (0%)	–
Gallbladder perforation	5 (41.7%)	6 (50.0%)	1 (8.3%)	0.185
Adhesions	39 (60.9%)	19 (29.7%)	6 (9.4%)	0.069
Distended GB aspiration	3 (18.8%)	12 (75.0%)	1 (6.3%)	<0.001
Conversion to open surgery	1 (25.0%)	1 (25.0%)	2 (50.0%)	<0.001

The mean operative time that is from incision to dressing was 87.98 ± 29.73 minutes, with 75 (64.7%) patients having operative duration ≤90 minutes. The mean hospital stay was 2.42 ± 0.81 days, with 74 (63.8%) patients discharged within two days.

On stratification by GWT, severe gallbladder wall thickening (>7 mm) was strongly and independently associated with multiple markers of operative difficulty i.e., prolonged operative time (>90 min): 83.3% vs. 30.3% in normal GWT group (p < 0.001), distended gallbladder requiring aspiration: 66.7% vs. 3.9% (p < 0.001), conversion to open cholecystectomy: 33.3% vs. 1.3% (p < 0.001), subhepatic drain placement: 66.7% vs. 5.3% (p < 0.001), dense adhesions: 100% vs. 51.3% (p = 0.069, trend toward significance). Postoperative hospital stay was significantly longer in the severe GWT group (2.33 ± 0.8 days vs. 1.43 ± 0.6 days in normal GWT; p = 0.036) (Table-IV).

**Table-IV:** Comparison of gallbladder wall thickness with operative outcomes (n=116)

Operative outcomes	Normal (<3 mm)	Moderate (3–7 mm)	Severe (>7 mm)	p-value
Operative time (min)	77.5 ± 24.5	103.5 ± 23.0	131.8 ± 47.5	<0.001
Conversion to open (%)	1.3%	2.9%	33.3%	<0.001
Hospital stay (days)	1.43 ± 0.6	1.68 ± 0.6	2.33 ± 0.8	0.036

## DISCUSSION

The principal finding of this prospective study was that severe preoperative gallbladder wall thickening (>7 mm) on ultrasound is a highly specific marker of difficult laparoscopic cholecystectomy in patients with acute calculous cholecystitis operated within 96 hours of symptom onset. Despite comprising only 5.2% of the cohort, this subgroup experienced a markedly higher burden of intraoperative adverse events, including a 25-fold increase in conversion rate, prolonged operative time, and frequent need for aspiration or drain placement.

Our results are consistent with international literature but add important nuance. Several workers have previously established that GWT >3–4 mm predicts difficulty.^1^,^2^,^12^-^14^,^16^-^18^ However, the use of a higher threshold (>7 mm) for “severe” thickening identifies a smaller, distinctly high-risk population in whom the predictive power is exceptionally strong. This higher cut-off yields superior specificity and positive predictive value compared with the commonly used 3–4 mm threshold, making it particularly useful for resource allocation in low-middle-income settings.

In comparison with international literature i.e. Kokoroskos et al. (n=1089) which reported progressive worsening with increasing thickness, but did not separately analyse a >7 mm group.^1^ Another study by Alotaibi et al.^2^ used >3 mm cut-off and found significant associations, yet the magnitude of risk escalation in our >7 mm group exceeds most reported figures. Regional studies from Pakistan and neighboring countries have been limited and older;^17^ our contemporary data from a tertiary care University hospital confirm that the association remains valid in South Asian populations with potentially different inflammatory responses and delayed presentation patterns. A recent study published by Dwivedi et al also explored pre-operative GWT in patients with gall stones and its association with gall bladder carcinoma or precursor lesions with no significance.^20^

The new contribution of this work to medical literature lies in demonstrating that, even when laparoscopic cholecystectomy is performed early in acute calculous cholecystitis, a single ultrasound measurement of gallbladder wall thickness can reliably stratify patients into a large very low risk majority and a small ultra-high-risk minority. A reported GWT >7 mm identifies patients in whom severe inflammation has a high risk of complicating a safe total cholecystectomy. In comparison to a recent similar study published from another South Asian country which evaluated patients with GWT in two groups only that is less than and more than 3mm, findings were very similar as increased GWT was strongly associated with difficult LC with higher adhesion grades, increased operative time and intra-operative complications.^18^

This finding has immediate and practical clinical significance, particularly in low-middle-income settings. When GWT exceeds 7mm, surgeons and theatre teams should proactively plan for bail-out strategies such as fundus-first dissection, subtotal (fenestrating or reconstituting) cholecystectomy, liberal use of aspiration/decompression, and early conversion if critical view of safety cannot be achieved. It should also trigger automatic involvement of the most experienced available laparoscopic/ hepatobiliary surgeon (if available), and explicit preoperative counselling regarding higher risks of conversion, drain placement, and prolonged recovery. In institutions where senior laparoscopic expertise is not available round-the-clock which is a reality in many resource-constrained environments, this single, inexpensive, universally available parameter enables targeted triage and safer decision-making without requiring additional technology or investigations.

### Limitations:

It was a single center study with a small sample size for patients with severe GWT and hence further multi-center studies need to be conducted with larger sample size for severe GWT and those that compare GWT with symptom duration of patients.

## CONCLUSION

In conclusion, gallbladder wall thickness can be superior to symptom duration alone for predicting intraoperative difficulty in early acute cholecystitis. Routine reporting of exact GWT in millimeter in radiological reports can help surgeons to anticipate severe inflammation, prepare appropriate bail-out techniques, escalate expertise when needed, refer patient to a hepatobiliary center or wait and plan an interval cholecystectomy therefore ultimately improving patient safety and resource utilisation across diverse healthcare settings.

### Authors’ Contribution:

**NA:** Conception and design of the study, data acquisition, interpretation, analysis and manuscript writing

**AAS KS:** Data acquisition, interpretation, and manuscript writing

**ARA:** Conception and design of the study, overall supervision,

All authors have approved the final version of the manuscript and agree to be accountable for all aspects of the work.
